# Optimized lentiviral vector to restore full-length dystrophin via a cell-mediated approach in a mouse model of Duchenne muscular dystrophy

**DOI:** 10.1016/j.omtm.2022.04.015

**Published:** 2022-05-02

**Authors:** Jinhong Meng, Marc Moore, John Counsell, Francesco Muntoni, Linda Popplewell, Jennifer Morgan

**Affiliations:** 1 Dubowitz Neuromuscular Centre, Molecular Neurosciences Section, Developmental Neuroscience Programme, UCL Great Ormond Street Institute of Child Health, 30 Guilford Street, London WC1N 1EH, UK; 2 Department of Biological Sciences, School of Life Sciences and the Environment, Royal Holloway University of London, Egham Hill, Egham TW20 0EX, UK; 3 UCL Division of Surgery and Interventional Science, Charles Bell House, 43-45 Foley Street, London W1W 7TY, UK; 4 National Institute for Health Research, Great Ormond Street Institute of Child Health Biomedical Research Centre, University College London, London WC1N 1EH, UK

**Keywords:** lentivirus, Duchenne muscular dystrophy, native full-length dystrophin, sequence-optimized full-length dystrophin, myoblasts, muscle-specific promoter

## Abstract

Duchenne muscular dystrophy (DMD) is a muscle wasting disorder caused by mutations in the *DMD* gene. Restoration of full-length dystrophin protein in skeletal muscle would have therapeutic benefit, but lentivirally mediated delivery of such a large gene *in vivo* has been hindered by lack of tissue specificity, limited transduction, and insufficient transgene expression. To address these problems, we developed a lentiviral vector, which contains a muscle-specific promoter and sequence-optimized full-length dystrophin, to constrain dystrophin expression to differentiated myotubes/myofibers and enhance the transgene expression. We further explored the efficiency of restoration of full-length dystrophin *in vivo*, by grafting DMD myoblasts that had been corrected by this optimized lentiviral vector intramuscularly into an immunodeficient DMD mouse model. We show that these lentivirally corrected DMD myoblasts effectively reconstituted full-length dystrophin expression in 93.58% ± 2.17% of the myotubes *in vitro.* Moreover, dystrophin was restored in 64.4% ± 2.87% of the donor-derived regenerated muscle fibers *in vivo*, which were able to recruit members of the dystrophin-glycoprotein complex at the sarcolemma. This study represents a significant advance over existing cell-mediated gene therapy strategies for DMD that aim to restore full-length dystrophin expression in skeletal muscle.

## Graphical abstract

**Figure:**
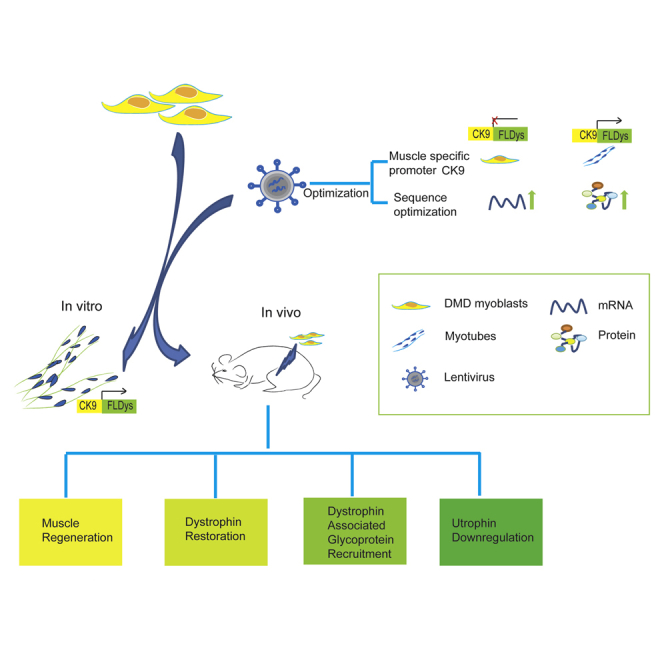

## Teaser

A lentiviral vector containing a muscle-specific promoter and sequence-optimized full-length *DMD* was developed. Functional full-length dystrophin is restored in the muscles of a DMD mouse model that has been transplanted with lentivirally corrected DMD myoblasts. This cell-mediated gene therapy strategy would be applicable to treatment of DMD patients.

## Introduction

Duchenne muscular dystrophy (DMD) is an X-linked genetic disorder caused by mutations within the *DMD* gene, leading to progressive muscle fiber necrosis and muscle wasting and weakness.^1^^,^^2^ Restoration of dystrophin protein in the affected muscles is the main therapeutic strategy for DMD. Adeno-associated viral (AAV) vectors coding mini- or microdystrophins are showing promising therapeutic effects in DMD clinical trials.^3^^,^^4^ However, these vectors are unable to deliver the full-length *DMD* cDNA, whose length of 11 kb is far beyond the 5 kb packaging capacity of AAVs; only a tri-AAV vector system can deliver full-length dystrophin, albeit at low efficiency.^5^^,^^6^ Although viral vectors with high packaging capacity, such as adenovirus,^7^, ^8^, ^9^ herpes simplex virus,^10^ foamy virus,^11^^,^^12^ or lentivirus,^13^ can accommodate the full-length *DMD* cDNA, the direct delivery of such vectors to skeletal muscles is challenging, as their bio-production scalability and myotropism remain suboptimal. Alternatively, a cell-mediated strategy can be explored to deliver the full-length dystrophin in DMD animal models.

Stem cell therapy is a potential treatment for DMD, as transplanted cells contribute to muscle regeneration and functionally reconstitute the muscle stem cell pool^14^^,^^15^ after their intramuscular injection in mouse models. But systemic delivery of stem cells to skeletal muscle remains challenging, due to the large number of cells required and inefficient targeting of skeletal muscle following intra-arterial or intravenous delivery. It has been suggested by patient groups^16^ that preserving or improving the function of the hand muscles of older DMD patients would be immensely beneficial to their quality of life. The thenar muscles of the hand control the fine movements of the thumb, including gripping, and would be key muscles that would benefit from dystrophin restoration. Although satellite cell-derived myoblasts are not systemically deliverable^17^ and have limited diffusion after local delivery,^18^, ^19^, ^20^, ^21^ they can still be considered to treat key muscles, such as thenar muscles, of DMD patients via intramuscular injection. Autologous stem cells genetically modified to express full-length dystrophin^12^^,^^13^^,^^22^ are preferable to allogeneic cells, as they are less likely to be rejected.^23^ We have previously shown that the full-length *DMD* cDNA can be packaged into a lentiviral vector^13^ and produce full-length dystrophin in myotubes differentiated from transduced myoblasts. However, the strategy requires further optimization and preclinical validation before progressing to clinical application.

In normal skeletal muscle, dystrophin is expressed in activated satellite cells^24^ and differentiated myofibers, but not in proliferating myoblasts.^25^ We have previously reported that expression of minidystrophin in DMD muscle stem cells can adversely affect their proliferation and myogenic differentiation *in vitro.*^26^ Therefore, it would be advantageous to use a muscle-specific promoter that drives transgene expression only in differentiated myotubes/myofibers and is small enough to fit into the lentiviral vector together with the large full-length *DMD* human cDNA.

The level of transgene expression is a key issue; many factors affect the transcription and translation of transgenes, and these can play an important role in delivering an effective therapy. To elicit a functional benefit^27^, ^28^, ^29^ within the treated muscle, the restored dystrophin protein level has to reach between 5% and 30% of normal dystrophin levels; it is better to have a lower level of dystrophin in the majority of fibers than a high level of dystrophin in a few fibers.^29^, ^30^, ^31^, ^32^, ^33^ In an effort to enhance expression, the full-length *DMD* cDNA was subjected to multiparametric sequence optimization, in which the native sequence was modified, with focus on GC content, codon optimization,^34^^,^^35^ mRNA transcription and stability, and protein translation. Sequence optimization of this nature has been used in the engineering of micro-^36^ and minidystrophin transgenes and has been successfully exploited in both a large animal model^37^ and clinical trials (NCT03375164 and GNT0004).^38^

A lentiviral vector containing a muscle-specific promoter and sequence-optimized full-length *DMD* transgene could constitute an effective cell-mediated gene therapy to treat all DMD patients, regardless of their *DMD* mutation.

## Results

### The CK9 promoter drives transgene expression predominantly in differentiated myotubes

**Figure 1:**
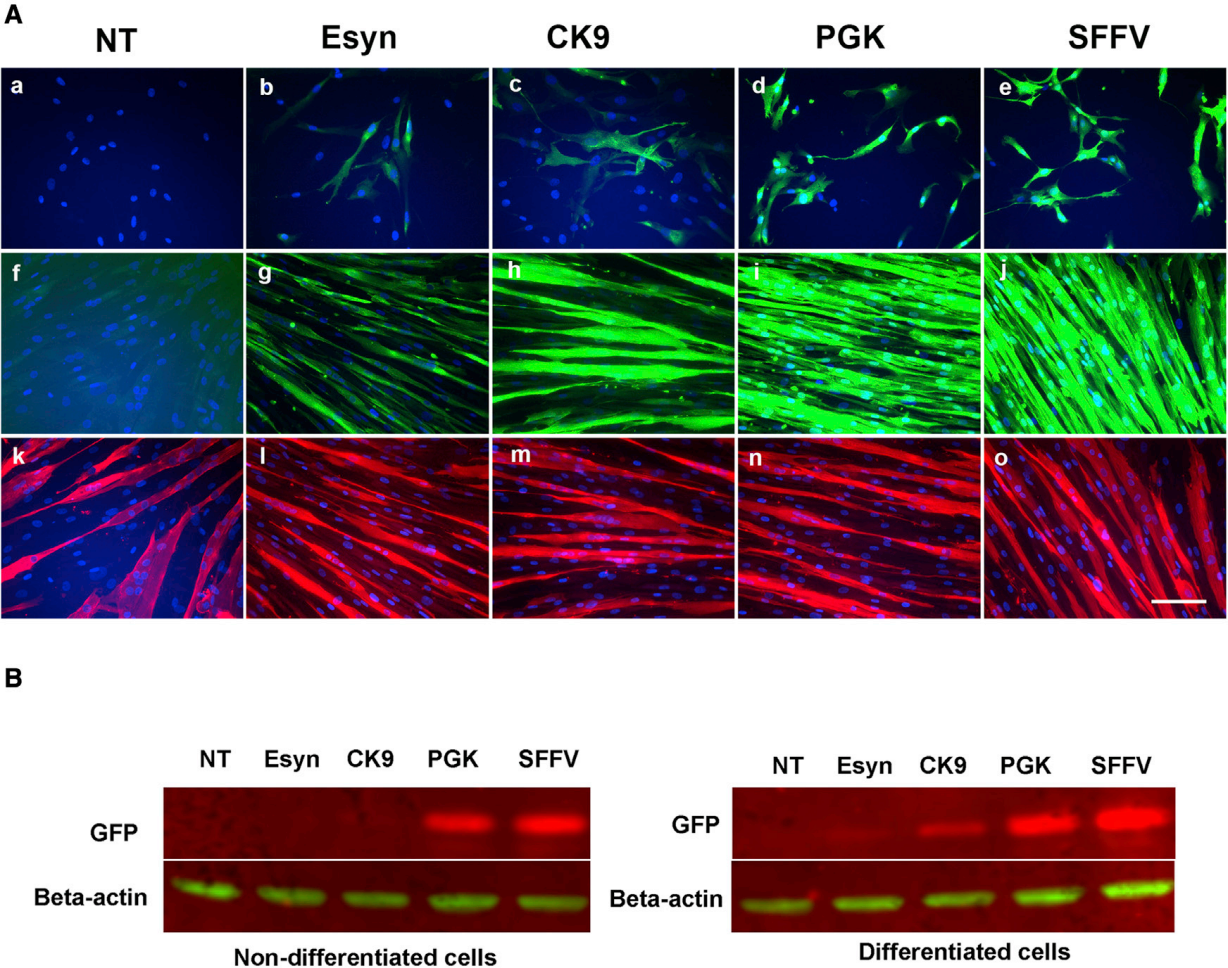
CK9- promoter drives transgene expression preferentially in differentiated myotubes DMD myoblasts (del Ex52) were transduced with lentivirus expressing EGFP driven by muscle specific promoters Esyn, CK9, or ubiquitous promoters PGK, SFFV. (A) shows the transgene expression (EGFP, green) in non-differentiated (a-e) or differentiated (f-j) cells. k-o shows the corresponding myosin heavy chain (MF20, red) staining of panels f-j. Nuclei were stained with DAPI (blue). Scale bar=50µm. (B) There was weak expression of the transgene in non-differentiated cells that were transduced by the lentivirus driven by Esyn or CK9, while the transgene expression increased dramatically in myotubes when these cells were induced to undergo myogenic differentiation. Between the two muscle specific promotors, CK9 drove stronger transgene expression than Esyn in myotubes. The transgene expression driven by PGK- or SFFV- was strong in both non-differentiated and differentiated cells. The levels of transgene expression driven by different promoters in non-differentiated and differentiated cells were confirmed by western blot analysis (B).

We transduced DMD myoblasts carrying an out-of-frame deletion of exon 52 (del Ex52) with lentiviruses expressing EGFP driven by a panel of promoters, to identify the optimal candidate for use in a lentiviral gene therapy context. In cells transduced with viral vectors containing an enhanced synthesized promoter (ESyn) (654 bp)^39^ or creatine kinase promoter 9 (CK9) (429 bp),^40^ there was little, if any, EGFP expression prior to myogenic differentiation, and EGFP was strongly expressed in differentiated myotubes (Figures 1A and 1B). In contrast, the majority of cells that were transduced with vectors driven by the phosphoglycerate kinase (PGK) or spleen focus-forming virus (SFFV) promoter had strong EGFP expression both pre- and post-myogenic differentiation (Figure 1A, a–e). These findings were confirmed by western blot (Figures 1B and S1A–S1C). Thus, we chose the CK9 promoter, the smaller of the two promoters that have increased expression after myogenic differentiation, for use in our modified lentiviral vector.

### Modification of the lentiviral vector by utilizing a muscle-specific CK9 promoter

**Figure 2:**
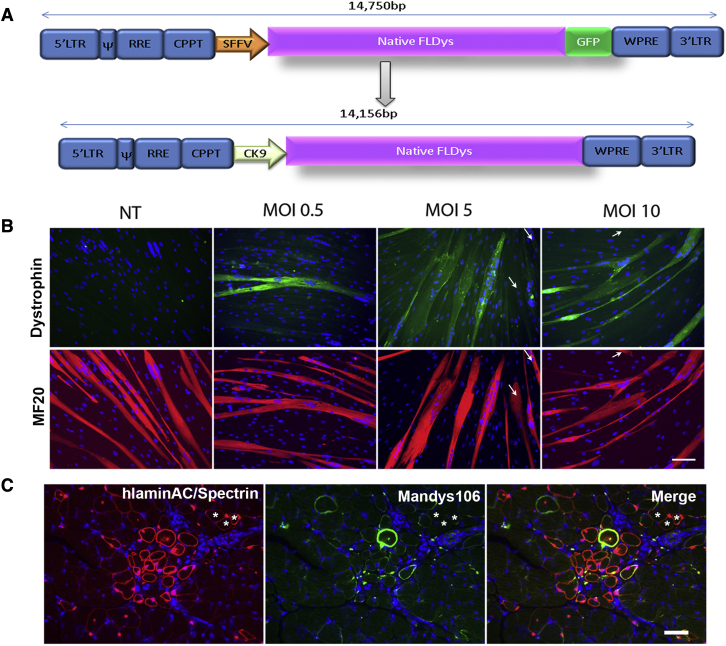
DMD myoblasts transduced with LV-CK9-nFLDys restored dystrophin expression *in vitro* and *in vivo* (A) Schematic illustration of the modified lentiviral vector (LV-CK9-nFLDys) modified from the previous version (LV-SFFV-nFLDys-EGFP): the EGFP cassette was removed and the SFFV promoter replaced by muscle specific promoter CK9-. (B) Myotubes (MF20, red) differentiated from DMD myoblasts transduced with different MOIs of the lentiviral vector expressed dystrophin (green). The majority of myotubes were dystrophin+ in cultures which were transduced with higher MOIs (≥5). White arrows point to the myotubes which were dystrophin negative. Nuclei were stained with DAPI (blue). Scale bar=100µm. (C) DMD myoblasts transduced with LV-CK9-nFLDys at MOI5 gave rise to human lamin AC/hSpectrin+ cells/fibres (red) in cryoinjured muscles of the mdx nude mouse, with a few donor derived fibres expressing dystrophin (Mandys 106, green) at their sarcolemma. ∗ indicate the donor myofibres which do not express dystrophin. Nuclei were stained with DAPI (blue). Scale bar=50 µm.

To make the lentiviral vector coding for full-length dystrophin more clinically compatible and to limit transgene expression to muscle, we modified the original full-length dystrophin vector^13^ by removing the EGFP cassette from the reading frame and replacing the SFFV with the CK9 promoter (Figure 2A), to produce the lentivirus LV-CK9-native full-length dystrophin (nFLDys; lentivirus in which expression of full-length dystrophin is driven by the CK9 promoter). *In vitro* assays of DMD myoblasts that were transduced with different MOIs of this lentivirus showed that increasing the amount of lentivirus (MOI >20) may have had an adverse effect on cell proliferation (Figure S2); thus, cells transduced with lower MOIs (0, 0.5, 5, or 10) were subsequently expanded and induced to undergo myogenic differentiation to evaluate the transgene expression. We found that dystrophin was present in the myotubes in all the transduced groups. While there were only a few dystrophin-positive myotubes in MOI 0.5-transduced cells, the majority of the myotubes in MOI 5- or 10-transduced groups contained dystrophin (Figure 2B). These results demonstrate that the titer of the lentivirus was high enough to produce dystrophin in the majority of the myotubes derived from the transduced cells (at MOI >5), without having to undergo an extra step of selection and enrichment after the transduction.

Next, DMD myoblasts that were transduced with LV-CK9-nFLDys at MOI 5 were transplanted into cryoinjured muscles of mdx nude mice^41^ to evaluate their contribution to muscle regeneration and dystrophin restoration *in vivo*. Donor fibers (human lamin AC^+^/human spectrin^+^) were present in muscles 4 weeks after transplantation. However, only 44.3% ± 4.31% of donor-derived muscle fibers expressed dystrophin, and this was at low levels (Figure 2C), indicating that further improvement of the lentiviral vector for better *in vivo* efficacy is necessary.

### Sequence optimization to improve the full-length dystrophin expression *in vitro*

**Figure 3:**
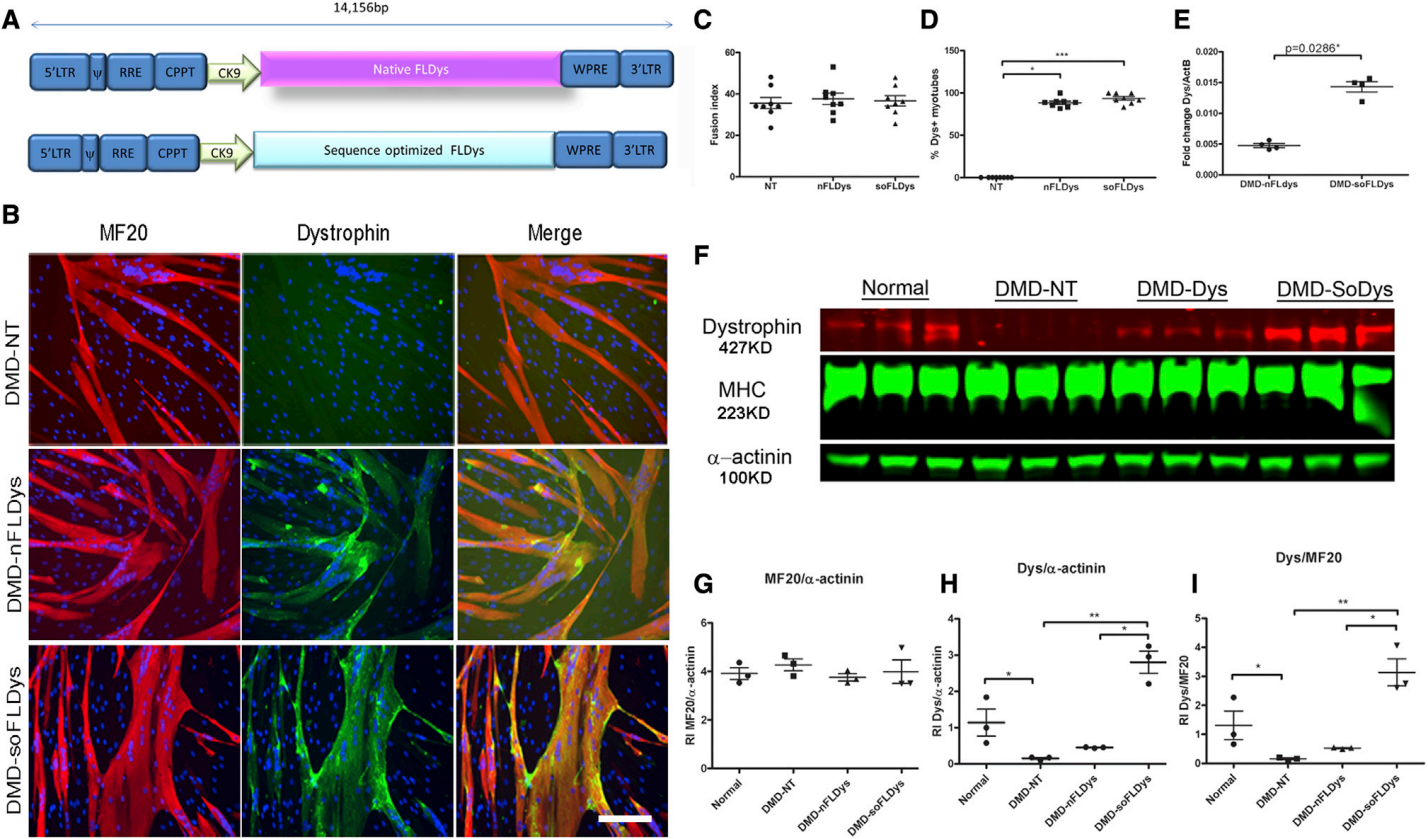
Sequence optimization of the full-length dystrophin resulted in higher transgene expression in transduced myotubes without affecting their myogenic capacity (A) Schematic illustration of the lentiviral vector further optimised by utilizing sequence optimized full-length *DMD* cDNA. (B) Immunostaining of dystrophin (green) and myosin heavy chain (MF20, red) on myotubes differentiated from DMD-NT, DMD-nFLDys and DMD-soFLDys myoblasts. Nuclei were stained with DAPI (blue). Scale bar=50µm. (C) Fusion index of the DMD-NT, DMD-nFLDys and DMD-soFLDys myoblasts showed that there were no differences in myogenic differentiation among these groups. (D) Percentage of the dystrophin+ myotubes in DMD-NT (0%), DMD-nFLDys (88.58±1.96%, p<0.05) and DMD-soFLDys (93.58±2.17%, p<0.001) groups. There were significantly more dystrophin+ myotubes in transduced groups than in non-transduced group. (E) RT-PCR analysis using cells differentiated for 5 days showed that there was significant higher (p=0.0286) full-length dystrophin transcript in DMD-soFLDys than DMD-nFLDys transduced cells, suggesting that sequence optimization increased dystrophin protein expression at both the transcriptional and translational level. F) Western blot using samples collected at D5 after the onset of myogenic differentiation from DMD-NT, DMD-nFLDys and DMD-soFLDys groups, to detect their expression of full-length dystrophin (427KD, red) and MF20 (223KD, green), using α-actinin (100KD, green) as loading control. Differentiated cells from normal myoblasts were also included as positive control. N=3 for each group. G, H and I show quantitative analysis of the dystrophin or MF20 expression in these groups. There was no difference in the MF20 expression between all groups (G), suggesting that the capacity of the DMD myoblasts to undergo myogenic differentiation was not affected by the lentiviral transduction, there was significantly higher expression of dystrophin in DMD-soFLDys than DMD-nFLDys transduced cells, using either α-actinin (H) or MF20 (I) as the normalization factor ∗p<0.05, ∗∗p<0.01. H).

To investigate whether the expression of dystrophin could be improved by sequence optimization, we produced lentiviruses encoding either sequence-optimized full-length dystrophin (soFLDys) or nFLDys, both driven by the CK9 promoter (Figure 3A), and transduced DMD myoblasts (del Ex52) at MOI 5. The viral copy number per cell in the transduced cell population was determined by qPCR, which showed 5.53 ± 0.12 copies for DMD-nFLDys and 4.97 ± 0.13 copies for DMD-soFLDys (Figure S9). After being induced to undergo myogenic differentiation, the fusion indices were 35.38% ± 2.68% (DMD- non-transduced [NT]), 37.65% ± 2.74% (DMD-nFLDys), and 36.47% ± 2.47% (DMD-soFLDys), with no statistically significant differences among these three groups (p > 0.05, one-way ANOVA), suggesting that the lentiviral transduction and the expression of dystrophin postdifferentiation had no adverse effect on the extent of differentiation (Figures 3B and 3C).

Next, we compared the full-length dystrophin expression at the transcriptional level, to determine if the sequence optimization would improve the transgene expression at the mRNA level. We performed qRT-PCR analysis of mRNA extracted from myotubes derived from DMD myoblasts (del Ex52) transduced with LV-DMD-nFLDys and LV-DMD-soFLDys, using primers specifically designed to recognize the common sequence of both nFLDys and soFLDys, but not the *DMD* transcript produced by the non-transduced DMD myoblasts (del Ex52), which lacks exon 52 (Figure S3). There was significantly higher (p = 0.0286, Student’s t test) *DMD* mRNA expression in cells transduced with LV-soFLDys than with LV-nFLDys (Figure 3E), suggesting that sequence optimization improved the full-length dystrophin expression at the transcriptional level.

Next, we investigated the expression of dystrophin in myotubes derived from transduced myoblasts. Immunostaining of dystrophin and MF20 (an antibody that recognizes the myosin heavy chain) showed that 88.58% ± 1.96% and 93.58% ± 2.17% of myotubes from DMD-nFLDys and DMD-soFLDys groups are positive for dystrophin, distributed in a punctate pattern along the myotubes (Figures 3B and 3D). In myotubes *in vitro*, dystrophin expression is sometimes punctate, especially when the dystrophin transgene is delivered to muscle precursor cells via viral vectors.^42^ This may be due to an uneven distribution of the dystrophin protein in differentiated myotubes, which are formed by the fusion of transduced and non-transduced cells. Western blot showed that the 427 kDa full-length dystrophin protein was present in normal, DMD-nFLDys, and DMD-soFLDys groups, but was absent, as expected, in the DMD-NT group (Figures 3F, 3H, and 3I). The extent of myogenic differentiation was similar in all groups, as indicated by the amount of MF20 expression in each group (Figures 3F and 3G). There were significantly higher amounts (around 6-fold higher) of full-length dystrophin expressed in DMD-soFLDys cells compared with DMD-nFLDys cells (p < 0.05, one-way ANOVA), when normalized to either α-actinin or MF20 (Table 1). There were around 40% and 240% of normal levels of dystrophin protein in the DMD-nFLDys and DMD-soFLDys groups, respectively.

**Table 1:** Relative expression of full-length dystrophin in transduced cells

	Normal	DMD-NT	DMD-nFLDys	DMD-soFLDys	Statistical comparison
Normalized to α-actinin	1.14 ± 0.37	0.15 ± 0.025 (background level)	0.45 ± 0.01	2.8 ± 0.31	p < 0.05, one-way ANOVA
Normalized to MF20	1.31 ± 0.49	0.16 ± 0.03 (background level)	0.53 ± 0.017	3.14 ± 0.46	p < 0.05, one-way ANOVA

In summary, our results show that DMD myoblasts transduced with LV-CK9-soFLDys resulted in higher dystrophin expression in myotubes at both the mRNA level (3.5-fold) and the protein level (around 6-fold) than in the same cells transduced with the same amount of LV-CK9-nFLDys, indicating that sequence optimization of the full-length dystrophin increases the *in vivo* restoration of the protein.

### Sequence optimization of the full-length dystrophin improves dystrophin restoration *in vivo*

We then investigated the contribution of the transduced cells to muscle regeneration and whether the sequence optimization could increase the amount of restored full-length dystrophin *in vivo*.

#### Dystrophin restoration in regenerated muscle fibers is greater in DMD-soFLDys-transplanted muscles

**Figure 4:**
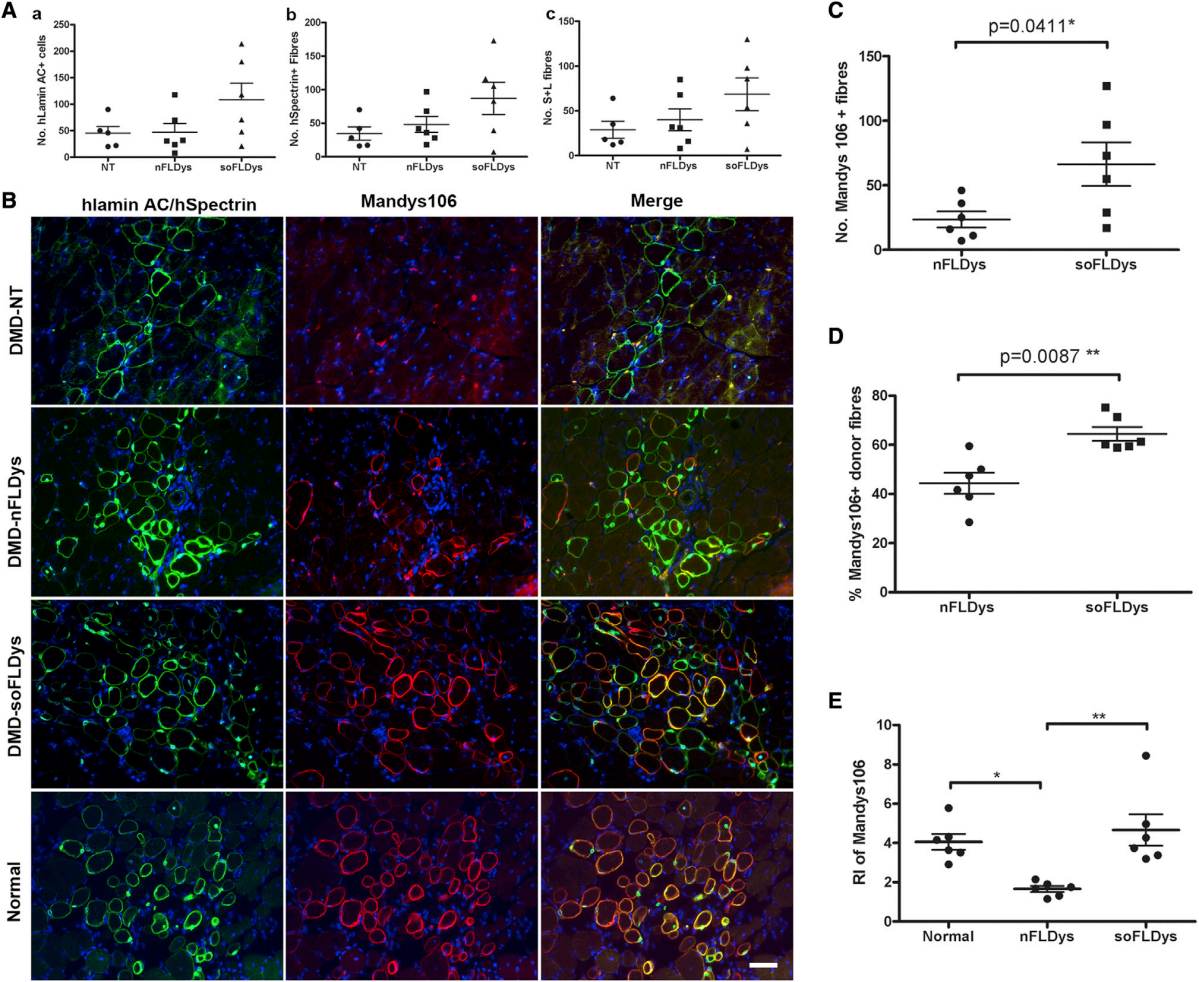
Restoration of dystrophin by lentivirally transduced cells *in vivo*. DMD-NT, DMD-nFLDys and DMD-soFLDys myoblasts were transplanted into cryoinjured muscles of mdx nude mice Muscle sections were stained for human laminAC (red), human spectrin (red) and human dystrophin (green) to determine the transplantation efficiency and the extent of dystrophin restoration in vivo. Muscles transplanted with normal myoblasts were used as control. (A) Transplantation efficiency of the DMD-NT, DMD-nFLDys and DMD-soFLDys were comparable, as determined by the number of human lamin AC+ nuclei (a), human spectrin+ fibres (b) and human spectrin+ fibres containing at least one human laminAC+ nucleus (S+L) (c). (B) Representative images of the donor fibres (human spectrin+, red) expressing dystrophin (Mandys106, green) in each transplantation group. Nuclei were stained with DAPI (blue). Scale bar=50µm. There were significantly more human dystrophin+ fibres (C) and a higher percentage of human dystrophin+/hSpectrin+ fibres (D) in DMD-soFLDys group than in DMD-nFLDys group. The relative intensity (E) of the human dystrophin was also significantly higher in DMD-soFLDys group than in DMD-nFLDys group, and the intensity of the human dystrophin was comparable to that of normal myoblast transplanted group. ∗p<0.05, ∗∗p<0.01.

First, we compared the transplantation efficiency within muscles that were transplanted with non-transduced or LV-CK9-nFLDys (MOI 5)- or LV-CK9-soFLDys (MOI 5)-transduced DMD myoblasts. There were no statistically significant differences in the numbers of either cells or myofibers of donor origin (numbers of human lamin AC^+^ nuclei, human spectrin^+^ fibers, or human spectrin^+^/human lamin AC^+^ fibers) between these groups (one-way ANOVA, p > 0.05) (Table 2 and Figures 4A–4C), indicating that the lentiviral transduction did not alter the engraftment capacity of the DMD myoblasts *in vivo*.

**Table 2:** Transplantation efficiency of cells in cryodamaged TA muscles of mdx nude mice

	DMD-NT (n = 5)	DMD-nFLDys (n = 6)	DMD-soFLDys (n = 6)	Statistical significance
Human lamin AC^+^ nuclei	45.6 ± 12.66	47.17 ± 16.13	108.7 ± 31.12	none (p > 0.05)
Human spectrin^+^ fibers	34.6 ± 10.02	48.17 ± 11.95	87.17 ± 24	none (p > 0.05)
S + L fibers	28.8 ± 9.7	40 ± 12.32	68.5 ± 18.26	none (p > 0.05)

Data are presented as the mean ± SEM; statistical analysis used one-way ANOVA, Kruskal-Wallis test followed by Dunn’s multiple comparison test.

Next, we investigated the restoration of the full-length dystrophin in muscles that were transplanted with different cells. As expected, in muscle sections of the DMD-NT group, there were no human dystrophin fibers (Figure 4B). There were significantly more (p < 0.05, t test) human dystrophin^+^/human spectrin^+^ fibers in muscles transplanted with DMD-soFLDys compared with DMD-nFLDys cells, which represents a significantly higher percentage (p = 0.0087, Student’s t test) of dystrophin-expressing donor fibers in the DMD-soFLDys group compared with the DMD-nFLDys group (Table 3 and Figure 4B). There was a significantly stronger expression (relative intensity) of human dystrophin in the normal (p < 0.05) and DMD-soFLDys (p < 0.01) groups compared with the DMD-nFLDys group, and no difference (p > 0.05) in the human dystrophin intensity between normal and DMD-soFLDys groups (one-way ANOVA) (Table 3 and Figures 4C, 4D, and 4E).

**Table 3:** Human dystrophin^+^ fibers in transplanted muscles

	DMD-nFLDys (mean ± SEM)	DMD-soFLDys (mean ± SEM)	Normal (mean ± SEM)	Statistical analysis
No. of human dystrophin^+^/human spectrin^+^ fibers	22.5 ± 6.3	55.33 ± 15.19	N/D	Student’s t test, p < 0.05
% human dystrophin^+^/human spectrin^+^ fibers	44.35% ± 4.31%	64.44% ± 2.87%	N/D	Student’s t test, p < 0.01
Relative intensity of human dystrophin	1.66 ± 0.15	4.67 ± 0.8	4.05 ± 0.40	one-way ANOVA, p < 0.05

N/D, not determined.

Our data show that, in comparison to DMD-nFLDys myoblasts, DMD-soFLDys myoblasts not only gave rise to a higher percentage of donor fibers that expressed dystrophin, but also had approximately 2.8 times stronger dystrophin expression in these donor fibers, after their transplantation into cryodamaged tibialis anterior (TA) muscles of mdx nude mice (Table 3, Figures 4C and 4D).

#### Higher level of α-sarcoglycan was recruited to the sarcolemma of dystrophin^+^ fibers in DMD-soFLDys myoblast-transplanted muscles

In DMD muscle, lack of dystrophin leads to loss of components of the dystrophin-glycoprotein complex (DGC) in muscle fibers,^43^, ^44^, ^45^ resulting in secondary pathological changes of the muscle.^46^, ^47^, ^48^ To achieve a better therapeutic outcome, both dystrophin and the DGC are required to be restored at the sarcolemma.^49^ In addition, recruitment of members of the DGC also serves as a functional readout for the restored dystrophin isoform within treated fibers.^50^, ^51^, ^52^, ^53^

We investigated the recruitment of the DGC protein α-sarcoglycan (α-SG) in donor-derived dystrophin-expressing muscle fibers, by co-immunostaining with Mandys 106 and α-SG on muscle sections of DMD-nFLDys, DMD-soFLDys, or normal myoblast-transplanted groups.

**Figure 5:**
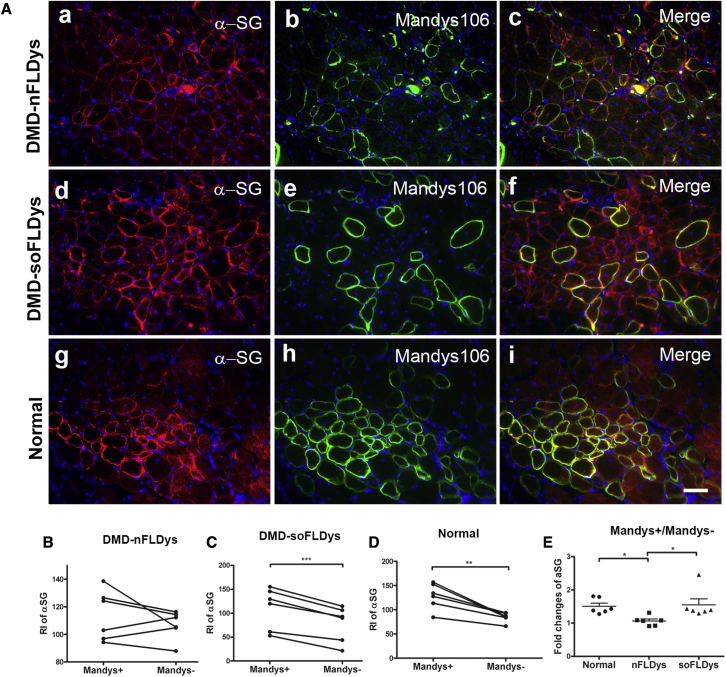
Increased expression of α-sarcoglycan (α-SG) in human dystrophin+ fibres in DMD-soFLDys, but not DMD-nFLDys myoblast-transplanted muscles (A) Muscle sections of DMD-nFLDys (a, b, c), DMD-soFLDys (d, e, f), or normal (g, h, i) myoblast transplanted groups were co-stained with antibodies recognizing human dystrophin (Mandys106, green) and α-SG (red). Nuclei were stained with DAPI (blue). Scale bar=50µm. The relative intensity of the α-SG in human dystrophin+ and human dystrophin- fibres within the same section was measured and compared using paired t-test (B, C and D). The fold change in the relative intensity of α-SG in human dystrophin+ fibres versus human dystrophin- fibres (Dys+/Dys-) among groups was compared using one-way ANOVA (E) In the DMD-nFLDys group, the level of α-SG in human dystrophin+ fibres was the same as that in human dystrophin- fibres (B), in DMD-soFLDys (C) and normal (D) groups, the expression level of α-SG was significant higher in human dystrophin+ fibres than in human dystrophin- fibres. The fold change in the relative intensity of α-SG in DMD-soFLDys group was equivalent to that of normal group (E). ∗p<0.05, ∗∗p<0.01, ∗∗∗p<0.001.

In the DMD-nFLDys group, the expression of α-SG was not increased in human dystrophin^+^ fibers (Figures 5A and 5B), and there was no statistically significant difference (paired t test, p > 0.05) in the intensity of α-SG between human dystrophin (Mandys 106)^+^ (113.9 ± 7.46) and human dystrophin^−^ fibers (106.8 ± 4.24) within the same section. In contrast, in the DMD-soFLDys group, the expression of α-SG was significantly higher (p < 0.001, paired t test) in human dystrophin^+^ fibers (110.8 ± 17.73) compared with human dystrophin^−^ fibers (78.24 ± 15.12) (Figures 5A and C), similar to the normal group, where the relative intensity of α-SG was also significantly higher (p = 0.0038, paired t test) in human dystrophin^+^ fibers (128.3 ± 10.91) compared with human dystrophin^−^fibers (84.7 ± 4.04) (Figure 5D).

The fold change of α-SG expression in human dystrophin^+^ fibers versus human dystrophin^−^ fibers in the DMD-nFLDys group (1.067 ± 0.059) was significantly lower (p = 0.0062, one-way ANOVA) compared with that of the DMD-soFLDys group (1.551 ± 0.1817) and the normal group (1.507 ± 0.095) (Figure 5E).

Our data show that, in myofibers derived from DMD-nFLDys myoblasts, the full-length dystrophin was not expressed at high enough quantities to restore the α-SG to levels detectable using immunohistochemistry (IHC). The sequence-optimized dystrophin vector, however, restored α-SG to levels similar to those found in donor-derived myofibers in muscles transplanted with control (non-DMD) myoblasts. Similar to α-SG, γ-SG in human dystrophin^+^ fibers were also found in the DMD-soFLDys and normal groups, but to a lesser extent in DMD-nFLDys-transplanted muscles (Figure S4).

#### Utrophin is downregulated in human dystrophin^+^ fibers in DMD-soFLDys myoblast-transplanted muscles

**Figure 6:**
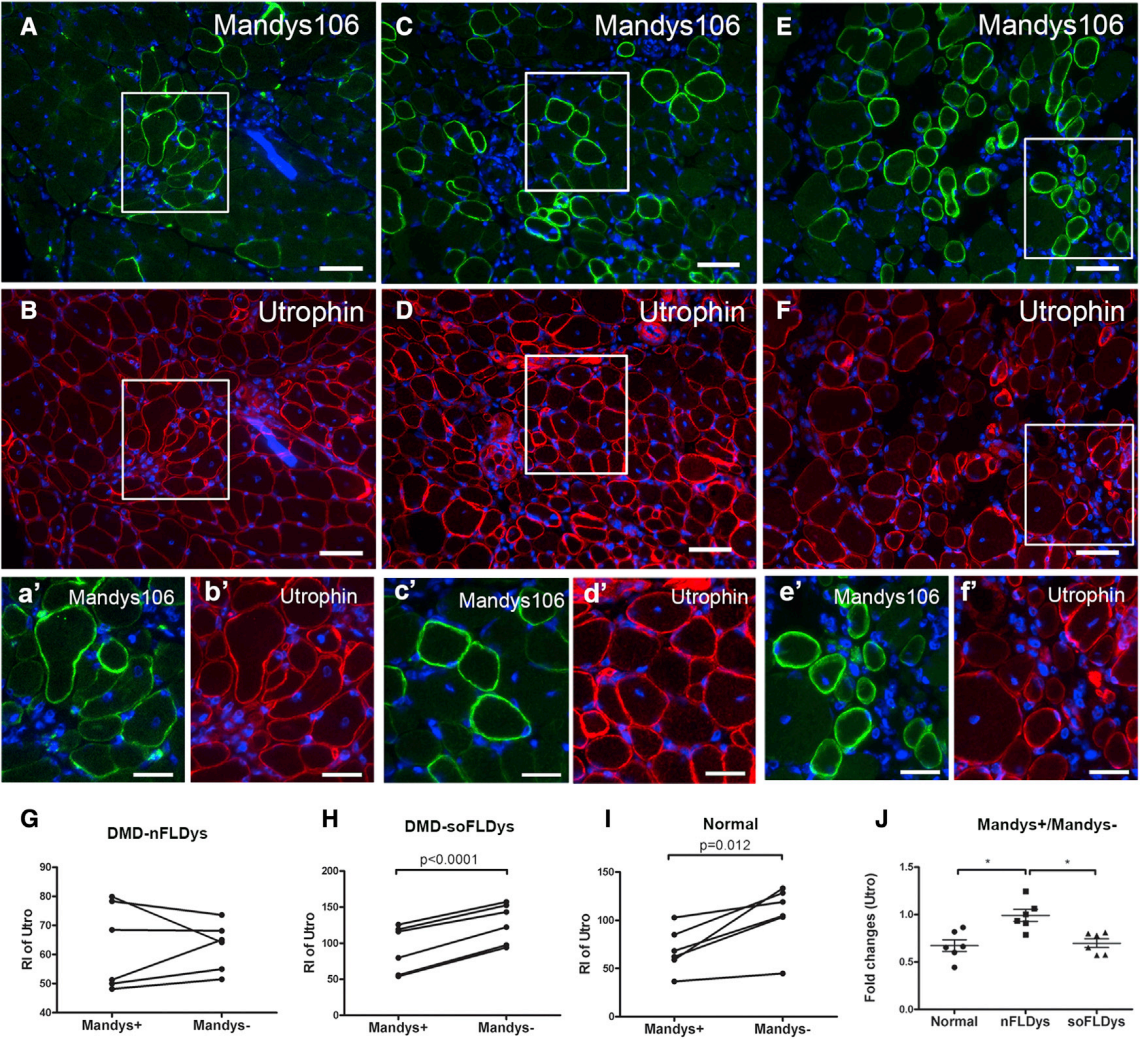
DMD-soFLDys myoblast-transplanted muscles contained donor derived muscle fibres which express sufficient full-length dystrophin to reduce utrophin expression at the sarcolemma of the fibres Muscle sections of DMD-nFLDys (A, B, a’, b’), DMD-soFLDys (C, D, c’, d’) or normal (E, F, e’, f’) myoblast-transplanted groups were co-stained with antibodies recognizing human dystrophin (Manys106, green) and utrophin (red). Nuclei were stained with DAPI (blue). a’, b’, c’, d’, e’ and f’ are enlarged images of the square region in their corresponding images labelled in capital letters. Scale bar=25µm. The relative intensity of utrophin in human dystrophin+ and human dystrophin- fibres within the same section was measured and compared using paired t-test (G, H and I). The fold change of the relative intensity of utrophin in human dystrophin+ fibres versus human dystrophin- fibres among groups was compared using one-way ANOVA. In the DMD-nFLDys group, the expression level of utrophin in human dystrophin+ fibres was the same as that in human dystrophin- fibres (A, B, a’, b’), and there was no statistically-significant difference between the human dystrophin+ and human dystrophin- fibres (G), in the DMD-soFLDys group, the expression level of utrophin was significantly lower in human dystrophin+ fibres than in human dystrophin- fibres (C, D, c’, d’, H), similar to that of normal group (E, F, e’, f’, I). The extent of the utrophin reduction in human dystrophin+ fibres versus human dystrophin- fibres was similar in DMD-soFLDys and normal groups (J). ∗ p<0.05.

Utrophin is an autosomal homolog of dystrophin that is upregulated in dystrophin-deficient mouse muscles, partially compensating for the missing dystrophin.^54^^,^^55^ The reduced expression of utrophin in mdx myofibers in which dystrophin has been restored is an indication that the restored dystrophin is functional.^11^ To determine to what extent the full-length dystrophin delivered via lentivirally corrected DMD myoblasts could lead to utrophin reduction, we performed double immunostaining of utrophin (with an antibody that recognizes both mouse and human utrophin) and human dystrophin in sections of DMD-nFLDys, DMD-soFLDys, and normal myoblast-transplanted muscles (Figures 6A–6F and 6a′–6f′) and measured the intensity of the utrophin in human dystrophin^+^ or human dystrophin^−^ fibers in each section. We found that utrophin expression (relative intensity) was similar in human dystrophin^+^ (62.68 ± 5.99) and human dystrophin^−^ (62.90 ± 3.38) fibers in DMD-nFLDys myoblast-transplanted muscles (Figure 6G) (p > 0.05, paired t test). The ratio of the utrophin intensity in human dystrophin^+^ versus human dystrophin^−^ fibers of this group was 0.9911 ± 0.06349 (mean ± SEM, n = 6), providing evidence that there was no reduction of utrophin in human dystrophin^+^ fibers, suggesting the dystrophin in this group is not restored at high enough levels to reduce the utrophin expression at the sarcolemma. In contrast, utrophin expression was significantly reduced in human dystrophin^+^ fibers (91.76 ± 13.35) compared with human dystrophin^−^ (127.8 ± 11.27) fibers in the DMD-soFLDys group (Figure 6H) (p < 0.0001, paired t test), which is similar to the normal group (Figure 6I) (p = 0.012, paired t test), in which there was also significantly less utrophin in human dystrophin^+^ fibers (69.01 ± 9.304) compared with human dystrophin^−^ fibers (105.6 ± 13.13). The ratio of the utrophin intensity in human dystrophin^+^ versus human dystrophin^−^ fibers in the DMD-soFLDys group was 0.6984 ± 0.04572 (mean ± SEM, n = 6), which is similar to the normal group, 0.6736 ± 0.06212 (mean ± SEM, n = 6). This suggests that myoblasts transduced with DMD-soFLDys, but not DMD-nFLDys, could restore sufficient dystrophin at the sarcolemma of the donor-derived fibers to downregulate utrophin expression, to an extent similar to that of normal myoblasts.

## Discussion

The overarching aim of this study was to develop an effective therapeutic strategy to restore the fully functional, full-length dystrophin isoform Dp427 in dystrophic skeletal muscles. Ideally, any therapeutic strategy should be applicable to all patients, regardless of which mutation they have in their *DMD* gene. Although gene editing^56^, ^57^, ^58^ can precisely correct a *DMD* mutation^59^^,^^60^ and give rise to full-length dystrophin expression,^61^ this method is highly mutation dependent and likely inefficient for full cDNA repair, meaning different designs need to be developed and validated for patients with different *DMD* mutations. In contrast, viral vectors introduce the dystrophin coding sequence into cells and are potentially applicable to the majority of the patients with a wide spectrum of mutations. However, the potential immune response against the full-length dystrophin will still need to be considered, especially for patients with large deletions or with deletions removing specific protein domains.^62^ This may be lessened by the incorporation of a muscle-specific promotor to limit dystrophin expression to muscle.

AAV has been used to restore dystrophin *in vivo*,^3^^,^^4^ but it accommodates only the small, partially functional dystrophin microdystrophin,^63^ due to its small packaging capacity.^5^^,^^64^ As AAV has a low integration rate *in vivo*,^65^ dystrophin expression may diminish over time and as a consequence of cell turnover. Furthermore, despite a plethora of preclinical studies to circumvent preexisting immunity to AAV vectors and to achieve immune toleration permissive of AAV re-administration, utilizing approaches such as plasmapheresis,^66^ alternative and capsid engineered AAV serotypes,^67^^,^^68^ and modulation of the immune response,^69^, ^70^, ^71^ current AAV-based gene therapies are limited to a single administration. In contrast, lentiviral vectors integrate into the host genome, giving sustained transgene expression. They can transduce muscle satellite cells *in vivo*,^72^^,^^73^ which would provide long-term therapy for muscular dystrophies such as DMD that are characterized by ongoing myofiber necrosis. Their safety and efficacy have been demonstrated in clinical trials,^74^ either *ex vivo*^75^, ^76^, ^77^, ^78^ or *in vivo.*^74^^,^^79^ Also, lentiviral vectors can package full-length dystrophin.^13^

The bottleneck preventing the direct application of lentiviral vectors *in vivo* is the relatively low titer that can be achieved using current systems and the inefficient targeting of skeletal muscles after systemic delivery, due to the relatively larger size (∼100 nm) of lentivirus particles^80^^,^^81^ versus AAV particles (∼20 nm),^3^^,^^82^ which negatively affects the dissemination of viral particles through the vasculature. They may, however, be used in a cell-mediated strategy, by transducing autologous stem cells.^42^^,^^83^ The use of autologous stem cells should reduce immune rejection. We have shown that it is feasible to remove the GFP cassette from the lentiviral vector and, by doing so, the transduced cells do not have to undergo *in vitro* manipulation such as fluorescence-activated cell sorting, followed by extra rounds of cell expansion, which would reduce the myogenic capacity of the muscle stem cells and their engraftment efficiency.^84^, ^85^, ^86^

We did find some discrepancies in the expression of human spectrin and human dystrophin at the sarcolemma of regenerated muscle fibers in transplanted muscles (Figure S5). In our model system, donor cells repair segments of host myofibers,^84^ giving rise to mosaic fibers containing myonuclei of both mouse and human origin. The finding of human spectrin, but not human dystrophin, within a myofiber is not surprising, as the grafted cells contained both transduced and non-transduced cells, so some myonuclei of human origin would produce dystrophin and others would not. The presence of human dystrophin, but not human spectrin, may be due to either the different sensitivities of these two human-specific antibodies or, possibly, the fact that spectrin spreads farther from the nucleus than does dystrophin.

When the SFFV promoter was replaced by the CK9 promoter to drive the native form of dystrophin (nFLDys) cDNA in the lentiviral vector, the restored dystrophin was at lower than normal levels both *in vitro* and *in vivo*. However, when the insert was replaced by a sequence-optimized full-length dystrophin (soFLDys), in comparison to nFLDys, there was a 6-fold increase in dystrophin protein expression *in vitro* and a 2.8-fold improvement in the dystrophin intensity at the sarcolemma in donor fibers *in vivo*. This was consistent with a previous report^36^ that the sequence optimization of microdystrophin transgenes improves the expression of dystrophin in mdx mouse muscles following AAV2/8 gene transfer.

By sequence optimization, which largely introduces human codon bias, it was anticipated that the major increase in transgene expression would occur at the translational level. However, we showed that the dystrophin expression driven by soFLDys was improved, not only at the translational level, but also at the transcriptional level, as evidenced by proximally 3-fold higher soFLDys transcripts than nFLDys transcripts in transduced cells. This is in line with the previous finding^87^ that codon biases are results of genome adaptation to both transcription and translation machineries, and codon biases determine the transcription levels by affecting chromatin structures. Apparently, the additional sequence modifications employed in our design of the cDNA also played a role. The GC content of the transgene was increased to improve the mRNA stability and, subsequently, prolong the transcript half-life. Overall, due to the human codon bias, sequence alterations, and increased GC content, an increase at the transcript level was anticipated^87^^,^^88^ and observed.

Compared with normal myoblasts, the amount of dystrophin in myotubes derived from DMD-soFLDys myoblasts was 2.4-fold higher *in vitro*, while the intensity of dystrophin in host muscles that had been grafted with DMD-soFLDys myoblasts was equivalent to, not higher than, that in host muscles that had been transplanted with normal donor myoblasts (*in vivo*). The discrepancies in the extent of *in vitro* and *in vivo* upregulation of the dystrophin transgene require further investigation. There is no information in the literature on the activity of the CK9 promoter in muscles of different fiber types. But interestingly, the CK6 promoter, although it drives dystrophin expression in most skeletal muscles, does not seem to be active in the diaphragm,^89^ and the MHCK7 promoter is more active in the mouse soleus muscle than the quadriceps and gastrocnemius, as it is more highly expressed in slow (MyHC type I) and fast oxidative (MyHC type IIa) fibers.^90^ It should be noted that the mouse TA muscle (the recipient muscle for our cell injections) consists predominantly of type IIa and IIb fibers. A possible drawback in the use of the CK9 promoter is that it does not drive dystrophin expression prior to myogenic differentiation, which may compromise the function of donor-derived satellite cells^25^^,^^91^ and limit long-term effectiveness of cell-mediated gene therapy. The desmin promoter has been shown to be superior to mouse muscle creatine kinase or human cytomegalovirus promoters when used in a lentivirus to drive EGFP expression in mouse myoblasts or mouse muscle^92^ and is also expressed in non-differentiated myoblasts.^93^ But its size (1.7 kb) presents additional challenges for packaging into a lentiviral vector that also contains full-length dystrophin.

The levels of the DGC proteins α-SG and γ-SG, as well as the utrophin expression in the transduced myotubes, further confirm the advantages of sequence optimization of the full-length dystrophin. Without optimization, the α-SG, γ-SG, and utrophin levels were not changed in donor-derived muscle fibers, most likely due to the insufficient restoration of dystrophin at the sarcolemma, while in muscles transplanted with DMD-soFLDys, the α-SG intensity was 1.5-fold higher in donor fibers, similar to the muscle group that was transplanted with normal myoblasts, suggesting the effective recruitment of DGC proteins in donor fibers corrected by sequence-optimized lentivirus. Similarly, utrophin expression was not changed in DMD-nFLDys transplanted muscles, while its expression was significantly reduced on donor fibers derived from DMD-soFLDys and normal myoblasts.

We have used myoblasts rather than induced pluripotent cell (iPSC)-derived myogenic cells as the donor cells in these experiments, as it has been previously shown that human satellite cells or satellite cell-derived myoblasts transplanted into mouse muscle contribute to satellite cells, as well as to regenerated muscle fibers.^94^, ^95^, ^96^, ^97^, ^98^, ^99^, ^100^, ^101^, ^102^, ^103^ Although there has been no direct comparison of the engraftment efficiency of human iPSC-derived myogenic cells and human myoblasts following their transplantation into the same mouse model, myoblasts give rise to similar numbers of myofibers of donor origin (up to 150) compared with transplanted human iPSC-derived myogenic cells,^104^, ^105^, ^106^, ^107^ after their injection into mouse muscle. This number of myofibers expressing dystrophin would not be sufficient to give any functional benefit to the transplanted muscle; for this, the host environment, the cells, or the transplantation method would have to be optimized to give at least 5% of dystrophin throughout the majority of the myofibers in the treated muscle.^27^, ^28^, ^29^, ^30^, ^31^, ^32^, ^33^

The number of myoblasts that we transplanted into each muscle (5 × 10^5^) may limit the number of myofibers of donor origin, but this number of cells in a final volume of 5–10 microlitres, is the most that can be injected into a mouse TA muscle. There is evidence (from studies in mouse and monkey) that the number of transplanted myoblasts does affect the number of regenerated muscle fibers to which they contribute,^19^^,^^108^ but the volume of cells that can be injected into muscle is a limitation.

In summary, our work demonstrates the efficacy of a novel lentiviral vector to restore full-length dystrophin *in vivo*, mediated by autologous muscle stem cells. Such a strategy takes advantage of autologous stem cells and a lentiviral vector containing a tissue-specific promoter and soFLDys, which can be readily progressed to clinical application to treat key muscles of DMD boys by intramuscular transplantation of autologous cells. Our strategy could also be used as a supplement to other treatment options, such as exon-skipping and AAV-mediated gene therapy, to provide longer-term protection of muscle fibers. Future work should focus on comparing other promoters and optimized dystrophin sequences.

## Materials and methods

### Ethics

The work was performed under the NHS National Research Ethics: setting up of a rare diseases biological samples bank (biobank) for research to facilitate pharmacological, gene and cell therapy trials in neuromuscular disorders (REC reference no. 06/Q0406/33) and the use of cells as a model system to study pathogenesis and therapeutic strategies for neuromuscular disorders (REC reference no. 13/LO/1826).

Mice were bred and experimental procedures were carried out in the Biological Services Unit, University College London Great Ormond Street Institute of Child Health, in accordance with the Animals (Scientific Procedures) Act of 1986. Experiments were performed under Home Office license nos. 70/8389 and 2611161. Experiments were approved by the local University College London ethical committee prior to the license being granted.

### Maintenance and differentiation of human myogenic cell preparations

Three human myogenic cell preparations were used in this study. DMD myoblasts (delEx52)^11^^,^^13^ and normal myoblasts derived from a healthy donor were maintained on collagen I (1×; Sigma, Dorset, UK)-coated culture vessels in M10 medium, consisting of Megacell DMEM (Sigma, Dorset, UK) supplemented with 10% fetal bovine serum (FBS; Thermo Fisher, Paisley, UK), 2 mM glutamine (Thermo Fisher, Paisley, UK), and 5 ng/mL bFGF (Peprotech, London, UK). Cells were kept at low density (2.5 × 10^5^ cells/T75 flask) and split every 3–4 days. For myogenic differentiation, cells were seeded onto 0.1 mg/mL Matrigel (VWR, Lutterworth, UK)-coated four-well plates (Nunc; for immunostaining of myosin heavy chain and dystrophin) or six-well plates (for western blot sample collection) at a density of 5 × 10^4^ cells/cm^2^ in proliferation medium. Medium was changed into differentiation medium (M2; Megacell DMEM containing 2% FBS) 24 h later, to initiate myogenic differentiation. Seven days after the onset of differentiation, cells in four-well plates were fixed with 4% paraformaldehyde for 15 min at room temperature and processed for immunostaining. Cells in six-well plates were used for protein sample collection for western blot analysis as described below.

### Lentiviral transfer plasmids and viral production

EGFP-expressing lentiviral vectors driven by either muscle-specific promoters (ESyn^39^ or CK9^40^) or ubiquitous promoters (SFFV or PGK) were generated using a previously described protocol.^42^ These lentiviruses were transduced into DMD pericytes^41^ or DMD myoblasts^13^ at equivalent MOIs (MOI = 10), and the transduced cells were then induced to undergo myogenic differentiation. The expression of GFP was monitored by immunostaining or western blot analysis at day (D) 0 (non-differentiated) or D7 (differentiated) after differentiation.

The open reading frame (ORF) of either nFLDys or soFLDys driven by the CK9 promoter was cloned into a third-generation lentiviral transfer plasmid, pCCLsin.cPPT.WPRE, using NEBuilder HiFi DNA assembly. To produce LV-CK9-nFLDys and LV-CK9-soFLDys, the transfer plasmid, packaging plasmids (pMDLg/pRRE and pRSV-Rev), and envelope plasmid (pMD2.G) were co-transfected at a ratio of 4:2:1:1 into HEK293T cells. Supernatant was collected at 48 and 72 h after transfection and concentrated by ultracentrifugation at 23,000*g* for 2 h at 4°C. The lentiviral titer was determined in DMD myoblasts as described below.

### Transduction of human DMD myoblasts and lentivirus titration

Cells were plated in 24-well plates at a density of 1 × 10^4^ cells/well and transduced with different amounts of virus. Cells were changed into fresh medium 6 h after the virus was added. The transduced cells were then expanded in M10 medium for subsequent experiments.

For lentivirus titration, genomic DNA of the cells was extracted using a DNeasy blood and tissue kit (Qiagen, Manchester, UK) according to the manufacturer’s instructions. Viral copy number within the transduced cells was determined using the Primetime qPCR probe assay (Integrated DNA Technologies, Leuven, Belgium). The primers and probes used for qPCR were WPRE-forward primer, TGGATTCTGCGCGGGA; WPRE-reverse, GAAGGAAGGTCCGCTGGATT; WPRE-probe, CTTCTGCTACGTCCCTTCGGCCCT; β-actin-forward primer, CAGCGGAACCGCTCATTGCCAATGG; β-actin-reverse primer, TCACCCACACTGTGCCCATCTACGA; and β-actin-probe, ATGCCCTCCCCCATGCCATCCTGCGT.

### Immunofluorescent staining of cells

Differentiated cells fixed by 4% paraformaldehyde (PFA) were immunostained using antibodies against GFP (rabbit polyclonal, 1:2,000; Thermo Fisher, Paisley, UK) or dystrophin (rabbit polyclonal, 1:1,000; Fisher Scientific, Loughborough, UK) and myosin heavy chain (MF20, mouse IgG 2b monoclonal antibody, 1:500; DSHB, Iowa City, IA, USA) at room temperature for 2 h, followed by Alexa 488-conjugated goat anti-rabbit IgG (H + L) (1:1,000; Thermo Fisher, Paisley, UK) and Alexa 594-conjugated goat anti-mouse IgG2b (1:1,000, Thermo Fisher, Paisley, UK) antibodies at room temperature for 1 h. Nuclei were stained with 10 μg/mL 4,6-diamidino-2-phenylindole (DAPI). Images were taken by an IX71 Olympus inverted fluorescence microscope. The fusion indices of the myotubes were calculated as the percentage of total nuclei within a field that was within an MF20^+^ myotube (containing at least three nuclei).

### Western blot

Cells transduced either before (D0) or after (D7) myogenic differentiation were lysed with radio-immunoprecipitation assay (RIPA) buffer (Sigma, Dorset, UK), supplemented with protease inhibitor (Roche, Welwyn Garden City, UK) on ice for 15 min. The cell lysate was boiled for 5 min and then centrifuged at 14,000*g* for 10 min at 4°C. Protein concentration was determined using a Pierce BCA protein assay kit (Thermo Fisher, Paisley, UK). Thirty micrograms per well of each sample was loaded onto a NuPAGE Novex 3%–8% Tris-acetate gel and run at a constant voltage of 150 V for 1.5 h, before being transferred to a nitrocellulose membrane using a constant current of 300 mA for 2 h. The membrane was then blocked with Odyssey block solution (LI-COR Biosciences, Cambridge, UK) for 1 h, before being incubated with primary antibodies against GFP (rabbit polyclonal IgG, 1:2,000; Thermo Fisher, Paisley, UK) or dystrophin (rabbit polyclonal IgG, 1:2,000; Fisher Scientific, Loughborough, UK) or MF20 (mouse monoclonal IgG2b, 1:1,000; DSHB, Iowa City, IA, USA), using α-actinin (mouse monoclonal IgG1, 1:10,000; Sigma, Dorset, UK) or tubulin 2.1 (mouse monoclonal IgG1, 1:3,000; Santa Cruz, Heidelberg, Germany) or β-actin (mouse monoclonal IgG1, 1:3,000; Sigma, Dorset, UK) as a housekeeping protein control. After being washed with PBS containing 0.1% Tween 20 (PBST) for 15 min × 3 at room temperature, the membrane was incubated with biotinylated anti-rabbit secondary antibody (1:1,000) for 2 h, followed by IRDye 680RD streptavidin and IRDye 800CW goat anti-mouse secondary antibodies (1:15,000; LI-COR Biosciences, Cambridge, UK) for 1 h at room temperature. The image of the blotted membrane was acquired by an Odyssey Clx infrared imaging system (LI-COR Biosciences, Cambridge, UK) using Image Studio Lite 5.2 software.

### qRT-PCR to determine the transcript of nFLDys and coFLDys in transduced cells

LV-nFLDys- or LV-soFLDys-transduced DMD myoblasts (del Ex52) with comparable viral copy number (VCN) were induced to differentiate into myotubes. Total RNA was extracted from cells at D5 of differentiation, using an RNeasy mini kit (Qiagen, Manchester, UK). This was treated with DNase I (Sigma-Aldrich, Dorset, UK) and subjected to a high-capacity cDNA synthesis reaction (Thermo Fisher, Paisley, UK), in accordance with the manufacturer’s protocols.

Quantitative PCR of the dystrophin expression was performed using the Primetime qPCR probe assay (Integrated DNA Technologies, Leuven, Belgium). The dystrophin primers and probes were designed to recognize a common sequence of nFLDys and soFLDys at exon 51 (forward) and exon 52 (reverse) of the dystrophin gene. Due to the lack of the exon 52 sequence in the myoblasts used in this study, the endogenous transcript will not be amplified; in this manner we are assessing only differences in transgene expression. The sequences of the dystrophin primers are Dys/soDys forward, TGAAAAACAAGACCAGCAA, and Dys/soDys reverse, GATATCAACGAGATGATCATCAAGCAGAA. However, different probes were used to detect the PCR product from DMD-nFLDys and DMD-soFLDys cells. The nFLDys probe was TGGGCAGCGGTAATGAGTTCTTCC, and the soFLDys probe was: AGCTGGAAGAACTGATCACAGCCG.

We also performed qRT-PCR using primers/probe against MYH1, as a control to monitor the extent of myogenic differentiation, and primers/probe against β-actin as the loading control. The sequences were MYH1 forward, GGTCGCATCTCTACGCCAGG; MYH1 reverse, ACTTTCGGAGGAAAGGAGCAG; and MHY1 probe, ATAACCTGCAGCCATGAGTTCCGA. The sequences of the primers/probe of β-actin are described above.

The relative amount of nFLDys transcripts in DMD-nFLDys cells is presented as the fold change between nFLDys and β-actin transcripts, calculated using the formula: 2ˆ-(delCt). DelCt refers to the differences in the cycle numbers between nFLDys and β-actin. The relative amounts of soFLDys transcripts in DMD-soFLDys cells were calculated in a similar manner and compared with those of nFLDys transcripts in DMD-nFLDys cells.

### Intramuscular transplantation

Four- to eight-week-old mdx nude mice^11^^,^^41^ were used as recipients for cell transplantation. On the day of transplantation, mice were anesthetized with isoflurane, and TA muscles were exposed and cryodamaged with three freeze-thaw cycles using a cryoprobe prechilled in liquid nitrogen.^84^^,^^109^ Cells (5 × 10^5^) in 5 μL medium were injected into each TA with a Hamilton syringe. Host muscles were cryoinjured immediately before cell transplantation, as we (and others) find that human myoblasts,^110^ human pericytes, and CD133^+^ cells^109^ and mouse satellite cells^14^^,^^111^ transplanted into non-injured muscles of immunodeficient, dystrophin-deficient mice do not engraft as well as they do following injection into injured muscles.

Grafted muscles were dissected 4 weeks after transplantation, mounted on corks in 6% gum tragacanth (Sigma, Dorset, UK), and frozen in isopentane prechilled in liquid nitrogen.

### Immunofluorescent staining on muscle sections

Transverse cryosections (10 μm) were air dried and blocked with AffiniPure F(ab′)_2_ fragment donkey anti-mouse IgG (H + L) (1:50; Jackson Immuno Research, Cambridge, UK) for 1 h at room temperature and stained with the following combinations of antibodies: (1) antibodies against human spectrin (mouse IgG 2b; Vector Labs, VP-S283, 1:100, Peterborough, UK), human lamin A/C (mouse IgG2b; Vector Labs, VP-L550, 1:500, Peterborough, UK), and human dystrophin (Mandys 106, mouse IgG2a, 1:200; Millipore). The numbers of human lamin A/C^+^ nuclei, human spectrin^+^ fibers, and human spectrin^+^ fibers containing at least one human lamin A/C^+^ nucleus (S + L) (as a confirmation that the spectrin^+^ fibers were of donor origin)^112^ were counted in representative transverse sections. The numbers of dystrophin^+^/human spectrin^+^ fibers were also quantified to evaluate the percentage of dystrophin-expressing fibers of donor origin. The intensity of the dystrophin (Mandys 106, red channel) on human spectrin^+^ fibers (green channel) was measured using MetaMorph software, normalized by the background intensity (we measured the intensity of the red channel on human spectrin^−^ fibers as background intensity) within the same muscle section, and compared among normal myoblast-, DMD-nFLDys-, and DMD-coFLDys-transplanted groups. (2) The second combination was dystrophin (Mandys 106, mouse IgG2a; Millipore) and α-SG (mouse IgG1, 1:100; Leica biosystems). (3) The third combination was dystrophin (Mandys 106, mouse IgG2a; Millipore) and γ-SG (rabbit polyclonal, 1:500; Santa Cruz). (4) The fourth combination was dystrophin (Mandys 106, mouse IgG2a) and utrophin (mouse IgG1, 1:200; Leica Biosystems). The results were acquired using a Leica microscope, and the intensity of α-SG, γ-SG, or utrophin on dystrophin^+^ or dystrophin^−^ fibers was quantified using MetaMorph software.

### Statistical analysis

For two-group comparisons, paired or unpaired Student t test was used. For comparisons involving three or more groups, one-way ANOVA (Kruskal-Wallis test) followed by Dunn’s multiple comparison test was used to determine statistical significance. Results presented in this study are displayed as the mean ± SEM. GraphPad Prism 5.0 software was used for statistical analysis and graph design. ∗p < 0.05, ∗∗p < 0.01, and ∗∗∗p < 0.001.

## Supplemental information

Document S1. Figures S1–S9

Document S2. Article plus supplemental information
