# Supplementary material for: Optimized lentiviral vector to restore full-length dystrophin via a cell-mediated approach in a mouse model of Duchenne muscular dystrophy
Source: Mol Ther Methods Clin Dev. 2022 May 2;25:491–507. doi: 10.1016/j.omtm.2022.04.015 (PMC9121076; doi:10.1016/j.omtm.2022.04.015)
Supplement: Document S1. Figures S1–S9 [file mmc1.pdf]

OMTM, Volume *25*

Supplemental information

Optimized lentiviral vector to restore full-length dystrophin via a cell-mediated approach in a mouse model of Duchenne muscular dystrophy

Jinhong Meng, Marc Moore, John Counsell, Francesco Muntoni, Linda Popplewell, and Jennifer Morgan

Sequence of ESyn promoter (547bp):

ccactacgggtctaggctgcccatgtaaggaggcaaggcctggggacacccgagatgcctggttataattaacccacaac
acctgctgcccccccccccccaacacctgctgcctgagcctgagcggttaccccaccccggtgcctgggtcttaggctctg
tacaccatggaggagaagctcgctctaaaaataaccctgtccctggtggatcggccgtccgccttcggcaccatcctcacg
acacccaaatatggcgacgggtgaggaatggtggggagttatttttagagcggtgaggaaggtgggcaggcagcaggtg
ttggcgctctaaaaataactcccgggagttatttttagagcggaggaatggtggacacccaaatatggcgacggttcctcac
ccgtcgccatatttgggtgtccgccctcggccggggccgcattcctgggggccgggcggtgctcccgcccgcctcgataa
aaggctccggggccggcggcggcccacgagctacccggaggagcgggaggcgccaagctctaga

Sequence of CK9 promoter (429bp):

Tgcccatgtaaggaggcaaggcctggggacacccgagatgcctggttataattaacccagacatgtggctgcccccccc
cccccaacacctgctgcctgctaaaaataaccctatgttcccggcgaagggccagctgtcccccgccagctagactcagc
acttagtttaggaaccagtgagcaagtcagcccttggggcagcccatacaaggccatggggctgggcaagctgcacgcct
gggtccggggtgggcacggtgcccgggcaacgagctgaaagctcatctgctctcaggggcccctccctggggacagcc
cctcctggctagtcacaccctgtaggctcctctatataacccaggggcacaggggctgccctcattctaccaccacctccac
agcacagacagacactcaggagccagccag

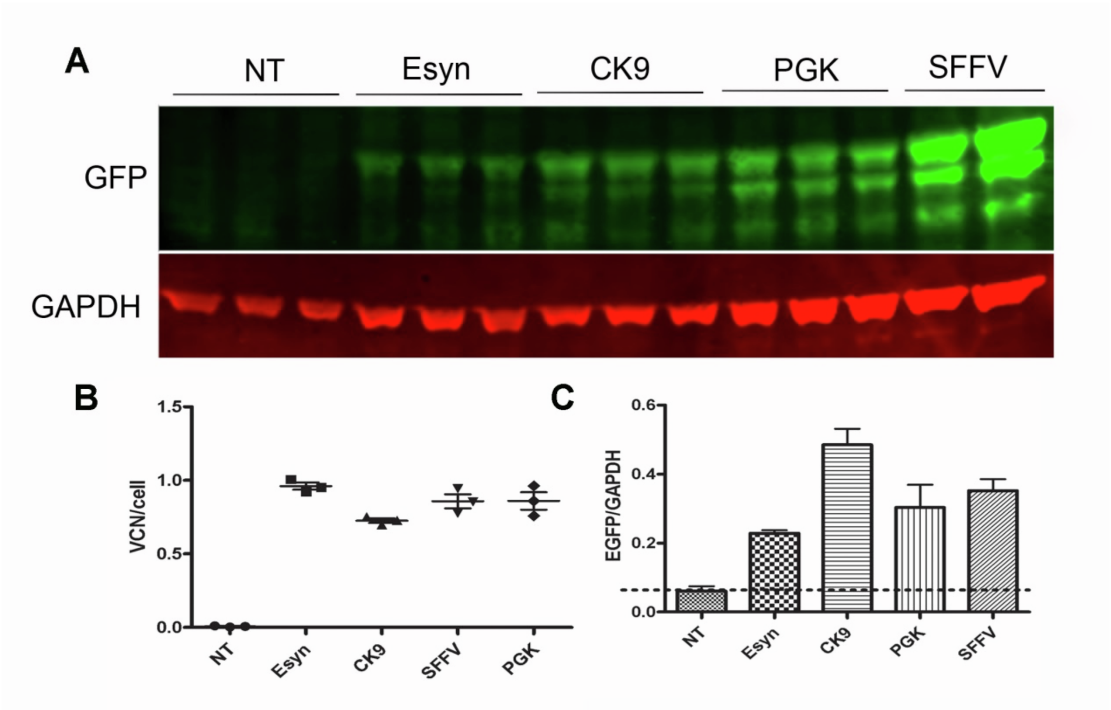

Figure S1. **EGFP expression in differentiated myotubes derived from DMD myoblasts which were transduced with lentivirus with different promoters.** DMD myoblasts were transduced with EGFP-coding lentiviruses driven by various promoters at similar MOIs. qPCR analysis revealed that the transduced cells in each group contained similar viral copy numbers per cell (B). Western blot (A) using samples collected from myotubes differentiated from different groups showed that there was no statistically significant difference in the EGFP expression between any of the transduced groups (One way ANOVA followed by Dunn's multiple comparison test, $p>0.05$) (C). The NT group was not included in the statistical analysis, but used to show the background intensity (dashed line) of the EGFP in the membrane.

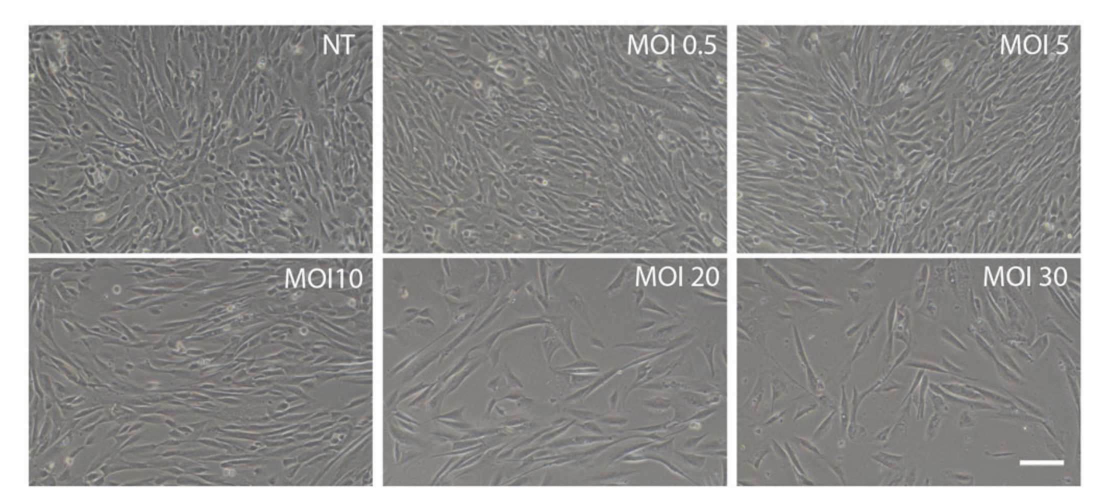

Figure S2. DMD myoblasts transduced with different MOIs of the LV-CK9-nFLDys showed a dose dependent effect on cell proliferation in high dose (MOI>10) transduced groups. Scale bar=100µm.

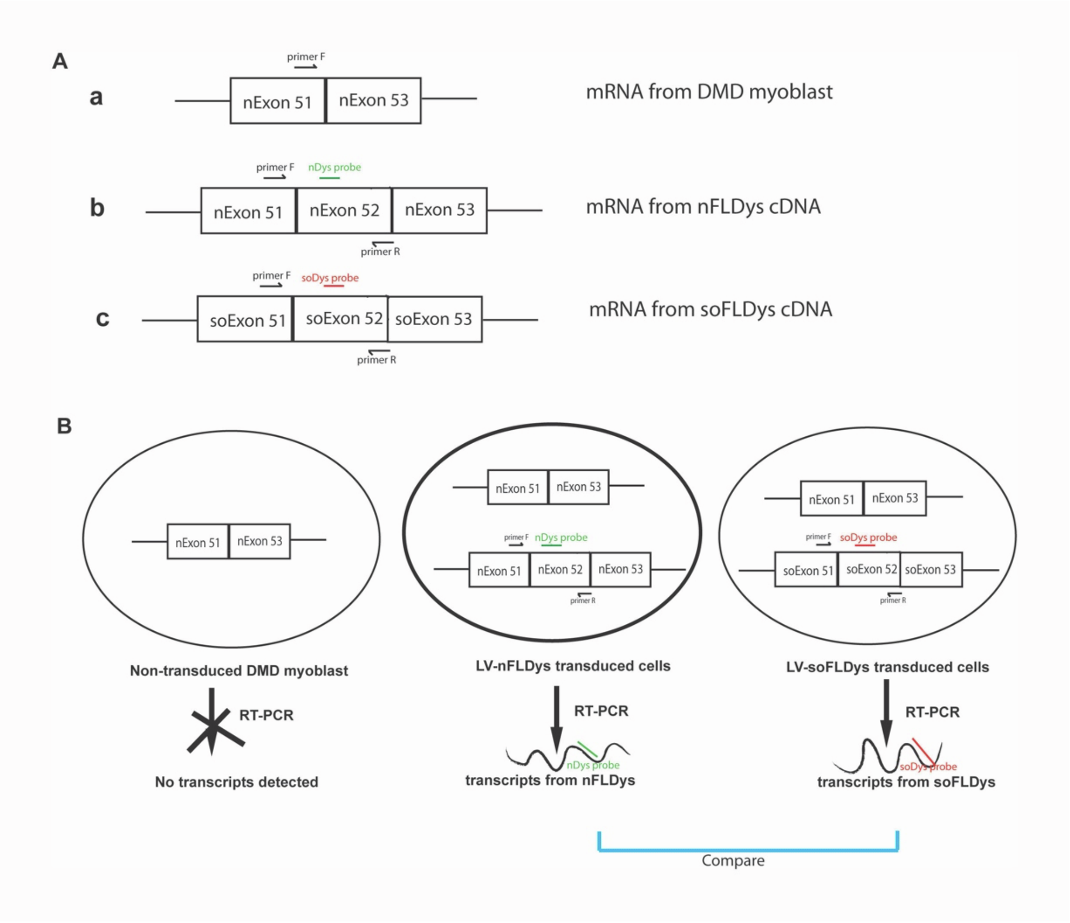

Figure S3. **Primer design for the qRT-PCR of soFLDys and nFLDys transduced cells.** The forward primer is located at the end of exon 51 of dystrophin, and the reverse primer is located at the end of exon 52. A). mRNA derived from DMD myoblasts (a), nFLDys cDNA (b) and soFLDys cDNA (c). B). In non-transduced DMD myoblasts, the dystrophin transcripts harbour an exon 52 deletion, thus there will be no amplicon produced with the Exon51-52 primer pair (due to the absence of the reverse primer binding site). In Lv-nFLDys or Lv-soFLDys transduced cells, the dystrophin transcript derived from the provirus contain exon 52, to which the primers bind, so after RT-PCR, a PCR product can be amplified that could be quantified and compared across samples.

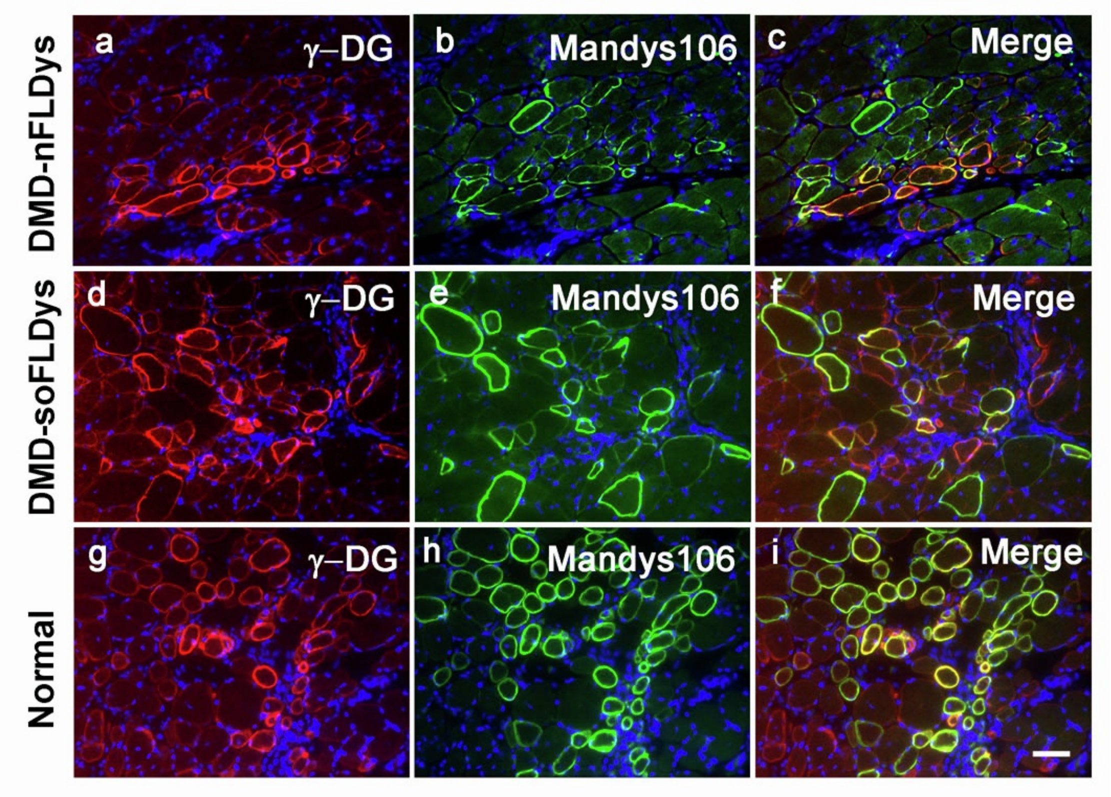

Figure S4. **Increased expression of γ-sarcoglycan (γ-SG) in Mandys106+ fibres in DMD-soFLDys, but not DMD-nFLDys myoblast-transplanted muscles.** A). Muscle sections of DMD-nFLDys (a, b, c), DMD-soFLDys (d, e, f) or normal (g, h, i) myoblast transplanted groups were co-stained with antibodies recognizing human dystrophin (Mandys106, green) and γ-SG (red). Nuclei were stained with DAPI (blue). Scale bar=50μm.

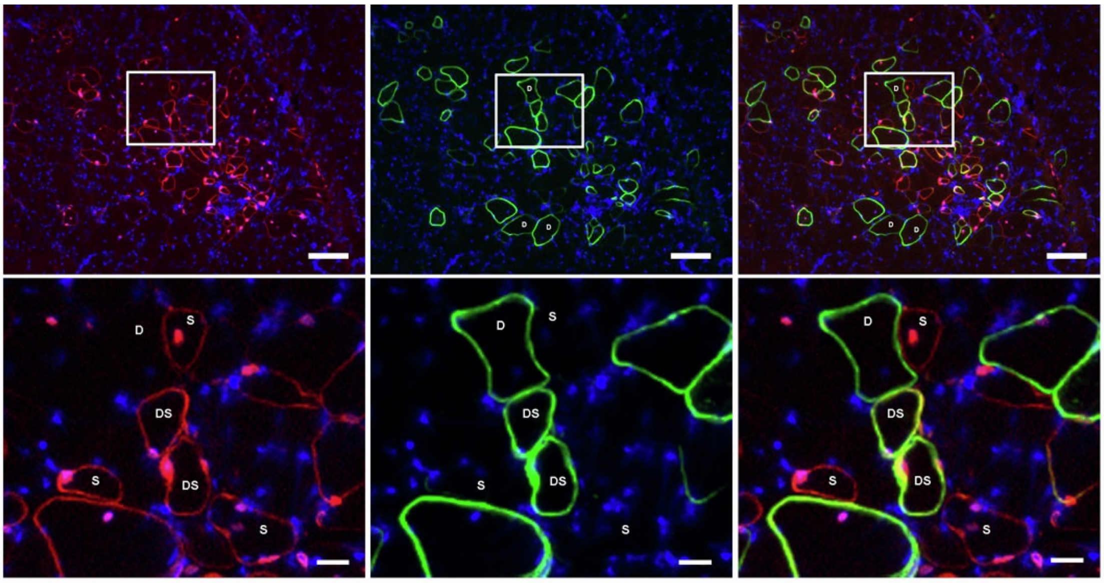

Figure S5. **Representative images showing that human spectrin (red) and human dystrophin (green) are not always both present on the same donor-derived myofibre.** Images are from a DMD-soFLDys myoblast transplanted muscle section. Upper panel shows the lower magnification images, scale bar=50µm. Lower panel shows the higher magnification images of the corresponding white box in the upper panel, scale bar=10µm. Nuclei are counter stained with DAPI (blue). D: Dystrophin+/Spectrin- fibre. S: Spectrin+/Dystrophin- fibre. DS: Dystrophin+/Spectrin+ fibre.

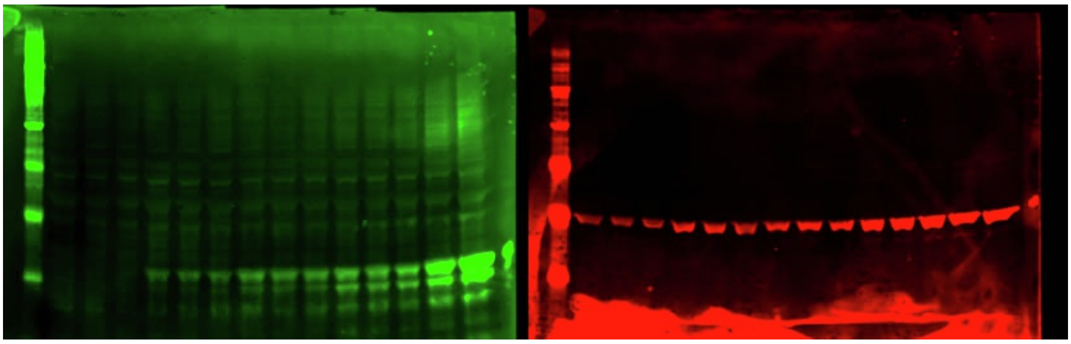

Figure S6. Original western blot image of Figure S1. Membrane was stained with GFP (green) and GAPDH (red).

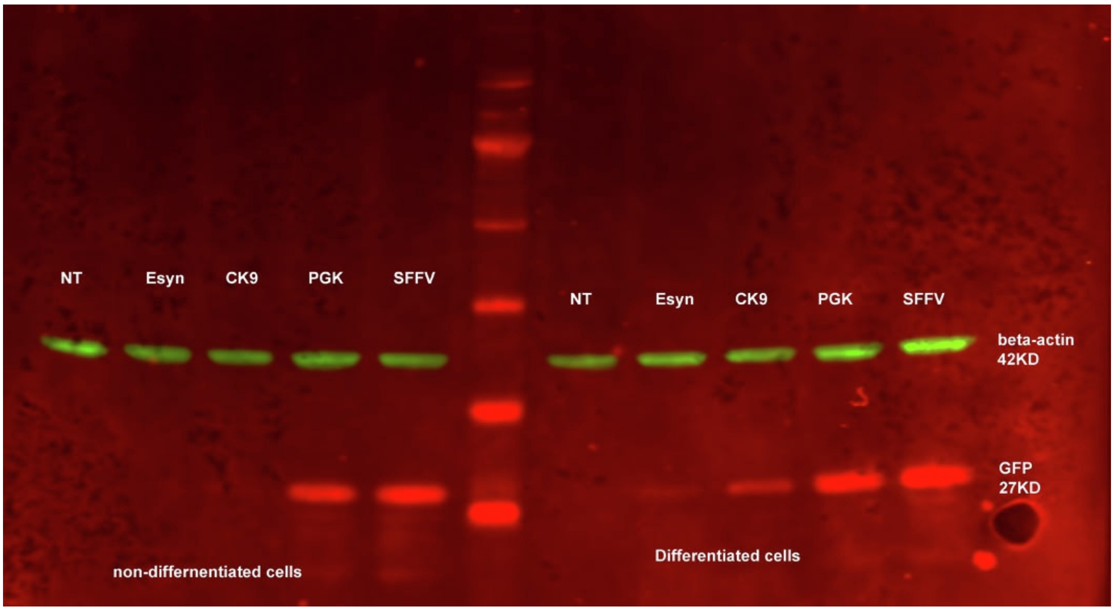

Figure S7. Original western blot image (Figure 1B in manuscript) of the DMD myoblasts that were transduced with EGFP- expressing lentiviruses driven by different promoters. Membrane was stained with GFP (red) and beta-actin (green). Left-hand sided image showed the non-differentiated cells, and right-hand sided image showed samples after undergoing differentiation.

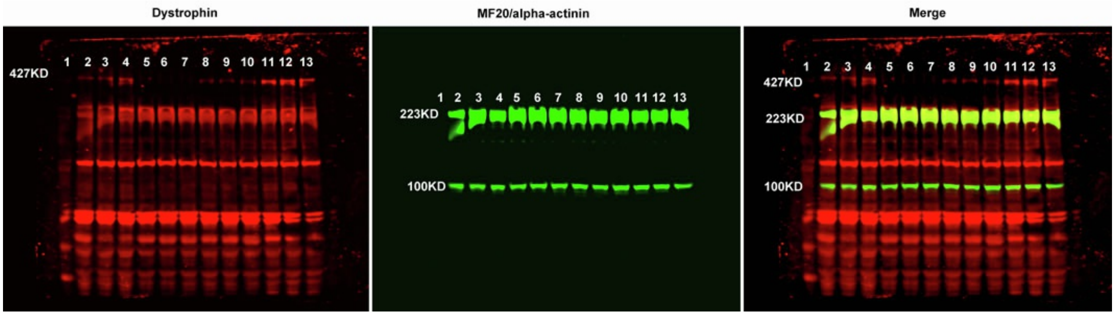

Figure S8. Original western blot images of the dystrophin (red), MF20/α-actinin (green) of myotubes derived from normal (lane 1-3), DMD (lane 4-6), DMD-nFLDys (7-9) and DMD-soFLDys (lane 10-12) myoblasts. (Figure 3 in manuscript).

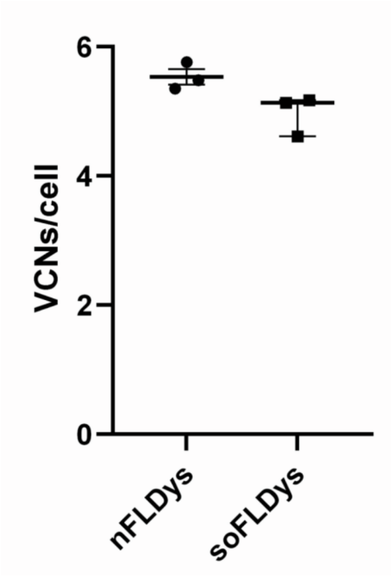

Figure S9. qPCR measurement of the viral copy numbers/cells in DMD-nFLDys and DMD-soFLDys cells used in the study.
